# Comparison of surgical versus medical termination of pregnancy between 13-20 weeks of gestation in Ethiopia: A quasi-experimental study

**DOI:** 10.1371/journal.pone.0249529

**Published:** 2021-04-01

**Authors:** Tesfaye Hurissa Tufa, Sarah Prager, Mekitie Wondafrash, Shikur Mohammed, Nicole Byl, Jason Bell

**Affiliations:** 1 Department of Obstetrics and Gynecology, Saint Paul’s Hospital Millennium Medical College, Addis Ababa, Ethiopia; 2 Department of Public Health, Saint Paul’s Hospital Millennium Medical College, Addis Ababa, Ethiopia; 3 University of Michigan Medical School, Ann Arbor, Michigan, United States of America; 4 Department of Obstetrics & Gynecology, University of Michigan, Ann Arbor, Michigan, United States of America; 1. IRCCS Neuromed 2. Doctors with Africa CUAMM, ITALY

## Abstract

**Background:**

Dilation and evacuation is a method of second trimester pregnancy termination introduced recently in Ethiopia. However, little is known about the safety and effectiveness of this method in an Ethiopian setting. Therefore, the study is intended to determine the safety and effectiveness of dilation and evacuation for surgical abortion as compared to medical abortion between 13–20 weeks’ gestational age.

**Methods:**

This is a quasi-experimental study of women receiving second trimester termination of pregnancy between 13–20 weeks. Patients were allocated to either medical or surgical abortion based on their preference. A structured questionnaire was used to collect demographic information and clinical data upon admission. Procedure related information was collected after the procedure was completed and before the patient was discharged. Additionally, women were contacted 2 weeks after the procedure to evaluate for post-procedural complications. The primary outcome of the study was a composite complication rate. Data were collected using Open Data Kit and then analyzed using Stata version 14.2. Univariate analyses were performed using means (standard deviation), or medians (interquartile range) when the distribution was not normal. Multiple logistic regression was also performed to control for confounders.

**Results:**

Two hundred nineteen women chose medical abortion and 60 chose surgical abortion. The composite complication rate is not significantly different among medical and surgical abortion patients (15% versus 10%; p = 0.52). Nine patients (4.1%) in the medical arm required additional intervention to complete the abortion, while none of the surgical abortion patients required additional intervention. Median (IQR) hospital stay was significantly longer in the medical group at 24 (12–24) hours versus 6(4–6) hours in the surgical group p<0.001.

**Conclusion:**

From the current study findings, we concluded that there is no difference in safety between surgical and medical methods of abortion. This study demonstrates that surgical abortion can be used as a safe and effective alternative to medical abortion and should be offered equivalently with medical abortion, per the patient’s preference.

## Background

Worldwide, termination of pregnancy is one of the most common procedures for reproductive aged women [1,2]. Abortion is safe when provided by a trained person using recommended techniques [3]. Despite limited evidence in developing countries, the safety of both surgical and medical methods of abortion in the second trimester is well demonstrated worldwide [4]. Both medical and surgical abortion have evolved to improve in effectiveness, acceptability, and rate of complications [5].

In countries with broadly accessible abortion services, most induced abortions occur in the first trimester [6]. Often, second trimester abortion is the only option available to women for many reasons, including but not limited to poor access to services and fear of disclosure of pregnancy [7–10].

Medical termination of pregnancy involves the use of medications (mifepristone and misoprostol) to cause the uterus to expel the pregnancy while surgical abortion involves preparing the cervix with mechanical or medical methods followed by dilation and evacuation (D&E) using instruments within the uterus [3,10].

Historical and contemporary studies have compared modern medical and surgical methods of termination of pregnancy resulting in a continued debate about the optimal method of second trimester abortion. Many studies show increased safety and effectiveness with surgical abortion, though others show a slight increase in the risk of fever requiring antibiotics with surgical abortion [11–14].

Motivated by the high rate of maternal death associated with unsafe abortion, Ethiopia adopted a legal reform in 2005 which expanded indications for safe abortion service [15]. Some of the indications in the new law include pregnancy as a result of rape or incest, fetal congenital abnormality, presence of physical and mental disabilities for the patient, and others [15]. Currently, the available working document in the country considers dilation and evacuation a second line of management in the second trimester, to be used only when there is a failed medical abortion [16].

Following the recent creation of a family planning fellowship program in a tertiary academic institution, St Paul’s Hospital Millennium Medical College (SPHMMC), started offering both medical and surgical abortion options up to 20 weeks’ gestational age. Even though D&E is considered by the national guideline as something to be offered only in cases of failed medical abortion, at SPHMMC D&E is offered equivalently to medication abortion for eligible patients. A review of 46 cases of D&E in the same institution demonstrated clinical safety with an overall complication rate of 2.3% [17]. This method of abortion newly introduced in the Ethiopian setting has a very low uptake, partly due to fear of acute complications and lack of robust evidence demonstrating effectiveness and safety in an Ethiopian context. Currently, no published data compare medical and surgical abortion in Ethiopia. This study aims to demonstrate the comparative safety and efficacy of medical and surgical abortion between 13–20 gestational weeks.

## Materials and methods

### Study design and study participants

A non-equivalent comparison group, quasi-experimental design was employed to include patients who were undergoing elective termination of pregnancy from 13–20 gestational weeks between November 1, 2018 and October 31, 2019. The study was conducted at Saint Paul’s Hospital Millennium Medical College (SPHMMC), a tertiary referral and teaching hospital in Addis Ababa, Ethiopia.

### Second trimester safe abortion standard of care in the institution

All patients with a gestational age between 13–20 weeks are fully counseled on the risks and benefits and given the option of medical or surgical abortion. Those who choose medical abortion receive mifepristone 200 mg orally on day 1 and are appointed to return to SPHMMC 24–48 hours later for admission and sublingual or vaginal misoprostol administration. Those who chose surgical abortion are given mifepristone 200 mg orally with or without intracervical laminaria and appointed to return the next day for surgical abortion. The decision to prepare the cervix with laminaria is made based on the gestational age. Those with advanced gestational age (>16 weeks) receive laminaria on the same day they receive mifepristone. Patients under 16 weeks gestational age received sublingual or vaginal misoprostol 400 mcg 2 hours prior to the procedure. Gestational age is determined by ultrasound using a composite calculation including the fetal parameters of head circumference, biparietal diameter and femoral length). The ultrasound findings are documented in the clinical chart. During the procedure, both medical and surgical abortion patients receive pain medication.

In the study, all women presenting for abortion care (medical or surgical) were approached by the study team to ascertain their willingness to participate in the study. The inclusion criteria for enrollment were, I) gestational age between 13–20 weeks confirmed with ultrasound, II) age ≥18 years, III) willingness to provide informed consent for the study, IV) having a working phone and willingness to be contacted for a follow-up questionnaire 2 weeks after the procedure. Failure to fulfill any of these inclusion criteria will automatically exclude participants from the study.

The study team provided interested patients with an overview of the study and the consent form, which they signed. At the time of initial arrival to the hospital, patients were counseled on the options of safe abortion (medical vs. surgical) using a standard counseling tool and allocated to either medical or surgical abortion groups based on their choice of procedure. Patients were interviewed by trained data collectors in their preferred language. A standardized questionnaire was written in English and translated into a local language (Amharic). Baseline sociodemographic and clinical information were collected upon admission to the hospital.

The remaining procedure-related information was collected after they recovered from the abortion procedure. Enrolled patients were followed throughout their time in the hospital for immediate complications or side effects related to the abortion procedure. Procedure-related information, including the decision for post-procedure observation, need and indication for additional intervention, and family planning related information, was abstracted from the provider’s note in the clinical chart. After discharge, patients were contacted via phone to answer follow-up questions.

### Ethics approval and consent to participate

Ethical approval was obtained from the Institutional review board (IRB) of Saint Paul’s Hospital Millennium Medical College and permission to perform the research was obtained from the head of the department of obstetrics and gynecology. The study was conducted after receiving an official letter of clearance from SPHMMC ethical review committee. Informed consent was obtained from all the surveyed women prior to the interview.

The primary outcome of the study was a composite complication rate, defined as the presence of one of the following complications: bleeding requiring observation, genital tract lacerations requiring repair, need for additional intervention to complete the abortion procedure, and one or more symptoms of pelvic infection at the time of follow up. We also assessed for the presence of other serious maternal complications including continued bleeding for more than two weeks, additional major surgery (laparotomy), and death. Also assessed were duration of hospital stay, degree of pain related to abortion procedure, acceptability of the procedure (as measured by patient satisfaction) and recommendation of the procedure to others. We based our sample size calculation on the composite complication rate of a previous cross-sectional study conducted in the same institution [17]. We assumed a clinically meaningful difference in composite complication rate of 15% between the groups with 80% power and a two-sided alpha of 0.05. Considering the low uptake of surgical abortion in the institution, we planned for a 3:1 ratio of medical versus surgical abortions. Given these assumptions, we needed at least 54 surgical and 162 medical abortion cases. Based on prior numbers of abortions performed in the hospital, we anticipated a one-year data collection would be sufficient to meet our sample size requirements.

The data were collected with open data kit then imported into Stata version 14.2 for data processing and analysis. Univariate analyses were performed using proportions and means (standard deviation), or medians (interquartile range) when the distribution was not normal. The different outcomes were compared between medical and surgical abortion procedure groups using Fisher’s exact test, independent t-test, or the non-parametric test of difference of means (Mann Whitney U test). A multiple logistic regression model was fitted to determine independent predictors of post-procedure complications while adjusting for measured confounding variables.

## Results

**Fig 1:**
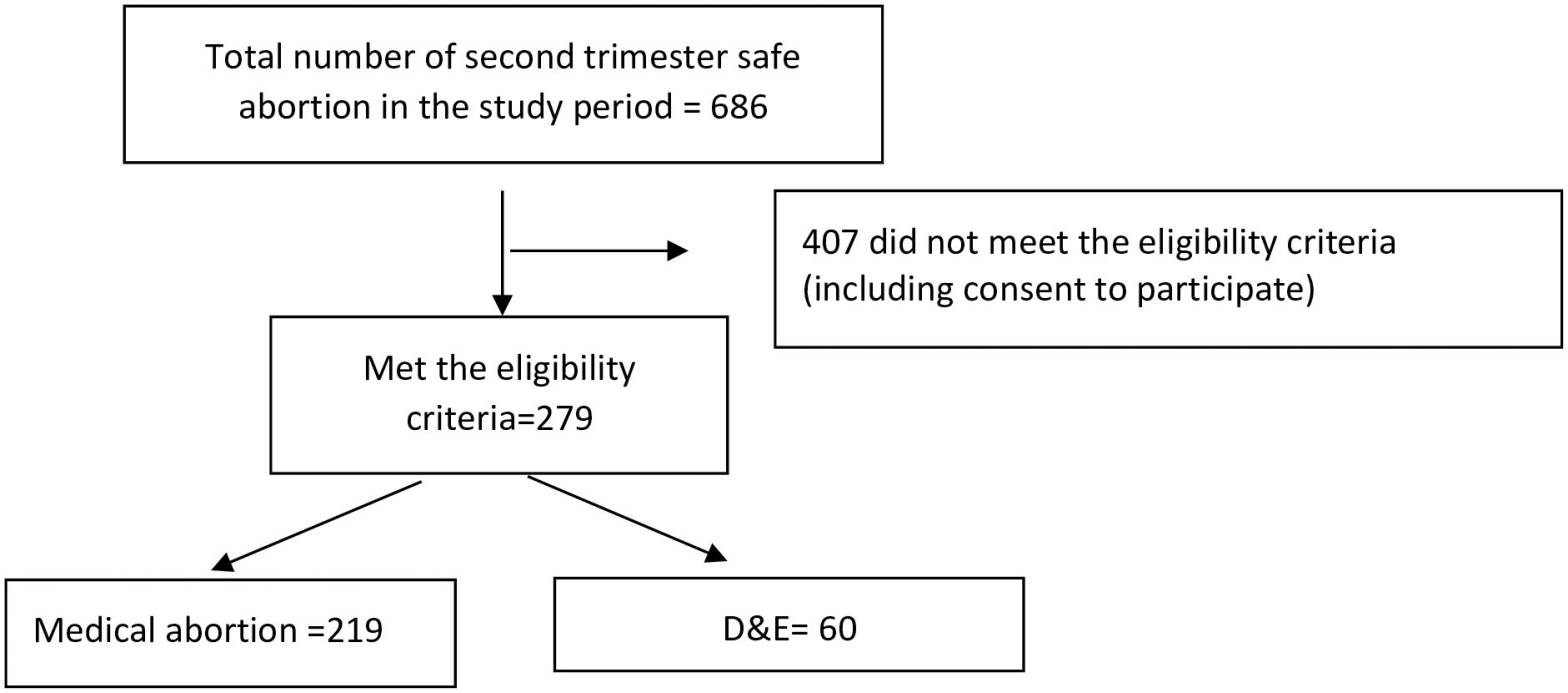
Flow chart of patient enrollment.

During the study period, a total of 279 women were included in the study, with 219 in the medical group and 60 in the surgical group (Fig 1). The baseline demographic characteristics for the two groups were similar except for age, place of residence, and prior delivery (Table 1). Significantly more patients in the surgical arm were older, live outside of Addis Ababa, and had a prior vaginal delivery (p <0.05).

**Table 1:** Baseline characteristics of study participants.

Characteristic	Level	Medical (n = 219)	Surgical (n = 60)	p-value
Age	18–25	176 (80.4%)	38 (63.3%)	0.008
	26–35	41 (18.7%)	19 (31.7%)	
	>35	2 (0.9%)	3 (5.0%)	
Religion	Muslim	34 (15.5%)	8 (13.3%)	0.92
	Orthodox	146 (66.7%)	41 (68.3%)	
	Protestant	39 (17.8%)	11 (18.3%)	
Income, median (IQR)		1000 (700, 2500)	1500 (1000, 2750)	0.14
Current residence	Addis Ababa	176 (80.4%)	38 (63.3%)	0.006
	Outside Addis Ababa	43 (19.6%)	22 (36.7%)	
Accompanying partner	No	180 (82.2%)	43 (71.7%)	0.071
	Yes	39 (17.8%)	17 (28.3%)	
Level of education	No formal education	18 (8.2%)	6 (10.0%)	0.42
	Grade 1–8 complete	91 (41.6%)	19 (31.7%)	
	Grade 8–12 complete	78 (35.6%)	22 (36.7%)	
	College graduate	32 (14.6%)	13 (21.7%)	
Gestational age (weeks)	13–15.6	68 (31.1%)	26 (43.3%)	0.20
	16–17.6	47 (21.5%)	11 (18.3%)	
	18–20.6	104 (47.5%)	23 (38.3%)	
Previous abortion	No	205 (93.6%)	52 (86.7%)	0.077
	Yes	14 (6.4%)	8 (13.3%)	
Prior delivery	No	160 (73%)	33 (55%)	0.008
	Yes	59 (27%)	27 (45%)	

One hundred eighteen (42.3%) women were referred from other health institutions, while the remaining 57.7 came to the hospital directly. Other clinical characteristics, including gestational age, were similar between the groups.

All patients in the medical arm received oral mifepristone 24–48 hours prior to misoprostol. Two hundred fifteen patients (98.2%) received misoprostol 400 micrograms every 3 hours, while four patients (1.8%) received a loading dose of 800 microgram misoprostol, followed by 400 micrograms every three hours. Sublingual was the most common route of administration for misoprostol (88.6%).

Ninety-eight percent (59/60) of patients in the surgical arm received mifepristone on day one. In addition to the mifepristone, surgical patients either received laminaria only (13.3%), misoprostol only (56.7%), or both laminaria and misoprostol (30.0%) for cervical priming prior to dilation and evacuation.

One hundred forty-four women in the medical arm (65.8%) received analgesia. Intramuscular (IM) diclofenac and oral ibuprofen were the most common analgesics used for medical abortions, accounting for 50% and 36.8% respectively. Other analgesics used were IM tramadol and oral paracetamol. All patients in the surgical arm received a paracervical block, and 53 (91.4%) additionally received IM diclofenac. Other pain medications used for surgical abortion were Intravenous (IV) diazepam and IM pethidine (Table 2).

**Table 2:** Pain medication used during surgical abortions.

Type of pain medication	Number	Percentage (%)
Paracervical block (PCB) only	7	11.7%
PCB + Diclofenac (IM)	37	61.7%
PCB + Diclofenac (IM) + Diazepam (IV) + Pethidine (IM)	10	16.7%
PCB + Diclofenac (IM) + Diazepam (IV)	5	8.3%
PCB + Diclofenac (IM) + Pethidine (IM)	1	1.7%

The composite complication rate among medical abortion patients is not significantly different than that of surgical abortion patients (15% versus 10%; p = 0.52). Nine patients (4.1%) in the medical arm required additional intervention (manual vacuum aspiration) to complete the abortion, while all patients in the surgical arm completed the procedure without a need for additional intervention (Table 3). More patients in the surgical arm were observed for bleeding (8.3% versus 1%; p< 0.002). Despite this, no patients in either arm required transfusion or any other medical or surgical intervention to control bleeding. There were no genital tract lacerations requiring repair in either arm and no maternal deaths reported during the study period.

**Table 3:** Procedure details and complications of study participants.

Factor	Medical (n = 219)	Surgical (n = 60)	p-value
Composite complication	33 (15%)	6 (10%)	0.52
Observed for bleeding	1 (0.5%)	5 (8.3%)	0.002
Symptoms of pelvic infection	21 (9.6%)	1 (1.7%)	0.055
Continued bleeding for two weeks	84 (38.3%)	21 (35%)	0.65
Duration of hospital stay (hours), median (IQR)	24 (12,24)	6 (4,6)	<0.001
Pain scale-medical-surgical			
No pain	0	5 (8.3%)	<0.001
Mild pain	48 (21.9%)	42 (70.0%)	
Moderate pain	117 (53.4%)	11 (18.3%)	
Severe pain	54 (24.7%)	2 (3.3%)	
Would recommend to others	184 (84.0%)	59 (98.3%)	0.003

We considered possible confounders and performed multivariable logistic regression to compare the composite complication rate between the medical and surgical arms and found no significant difference between the groups after controlling for measured confounders (Table 4).

**Table 4:** Multiple logistic regression analysis of composite complication.

Variables	Composite Complication* N (%)	Adjusted OR	95% CI
Method of abortion			
Medical	30 (83.3%)	1.00	
Surgical	6 (16.7%)	0.46	0.16–1.3
Age			
18–25	27 (75.0%)	1.00	
26–35	8 (22.2%)	1.44	0.49–4.2
> = 36	1 (2.8%)	2.64	0.22–32.2
Gestational age			
13–15.6	20 (55.6%)	1.00	
16–17.6	4 (11.1%)	0.22	0.07–0.72
18–20.6	12 (33.3%)	0.37	0.17–0.82
Previous abortion			
No	32 (88.9%)	1.00	
Yes	4 (11.1%)	2.39	0.6–9.1
Parity			
0	26 (72.2%)	1.00	
1	3 (8.3%)	0.26	0.06–1.1
> = 2	7 (19.4%)	1.01	0.32–3.2

*composite complication (observation for bleeding, one or more symptoms of infection, additional procedures required to complete the abortion). Those having one or more symptoms are considered as having complication (coded as; Yes = 1, No = 0).

Medical abortion was associated with a significantly longer median hospital stay (24 hrs., IQR 12–24 hrs.) as compared to surgical abortion (6 hrs., IQR 4–6 hrs.; p < .001). The surgical patients were more likely than the medical patients to recommend the same procedure to others (98.3% vs 84.0%; p = 0.003). Patients were also asked to score their satisfaction out of eight and mean satisfaction rate (scored from 0–8) between the groups is equivalent in the medical vs surgical groups (7.56 vs 7.90; p = 0.995).

**Fig 2:**
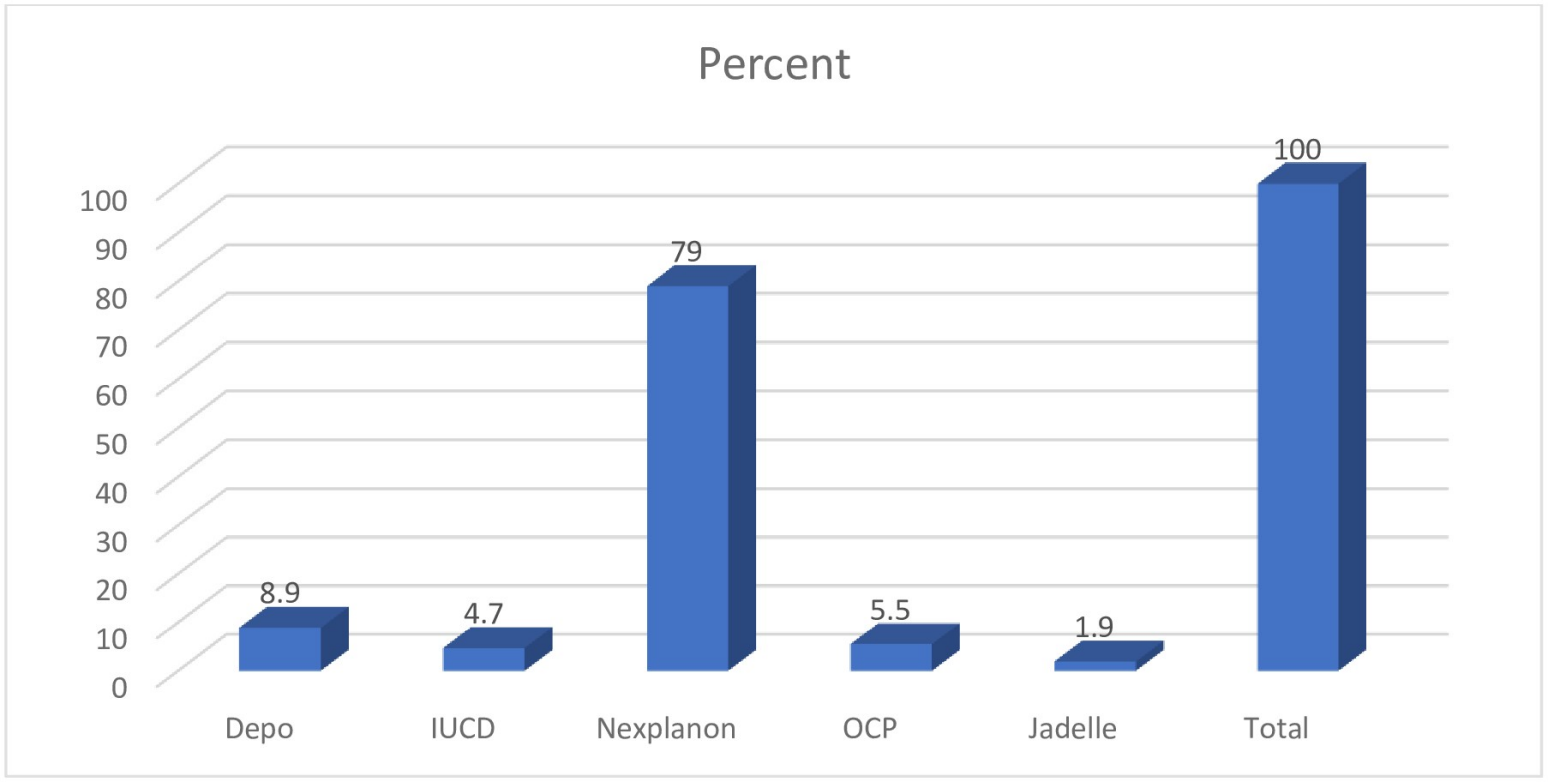
Post abortion family planning.

All patients in both the medical and surgical arms were counseled on family planning and 92% (257/279) received some form of family planning. The majority of the patients chose and received long-acting reversible methods, with Nexplanon, IUD and Jadelle contributing nearly 86% of the total contraception methods adopted (Fig 2).

## Discussion

The low complication rate in our study demonstrates that both medical and surgical abortion between 13–20 weeks can be safely practiced in an Ethiopian setting. Similar to other studies, the need for additional interventions to complete the abortion, occurred more frequently in the medical group [8,13,14,18–20]. At the time of the 2-week follow up enquiry, both ongoing bleeding and symptoms of pelvic infection were similar between the two groups.

All surgical abortion patients received prophylactic antibiotics and some form of pain medication during the abortion procedure. Conversely, only 65.8% of medical abortion patients received pain medication, which may explain the higher reports of moderate to severe pain in the medical as compared to the surgical arm. This reveals the need for standardization of pain management, especially for medical abortion. Additionally, further investigation is needed to identify the reasons for lower pain medication administration among women undergoing a medical abortion.

The median duration of stay is significantly shorter in the surgical abortion group. This finding is consistent with other studies [13,21]. Interestingly, patients who came from outside Addis Ababa were more likely to choose the surgical procedure, possibly to avoid the prolonged hospital stay associated with medical abortion. Similar to other studies, the mean satisfaction rate (scored from 0–8) between the groups is equivalent (7.56 vs 7.90; p = 0.995) [22,23]. Importantly, this study found that 42.3% of all patients were referred from nearby health institutions. As SPHMMC is striving to be a center of excellence for abortion care, it is good that nearby institutions know where/how to refer patients for abortion. Alternatively, this also indicates a need for capacity building at nearby health centers to reduce/avoid unnecessary delays in receiving care. Integrating D&E training into medical schools and residency programs is one way to build capacity and potentially address these delays. Finally, the high rate of family planning counseling and provision demonstrates the successful integration of family planning into abortion care at the institution.

### Strengths and limitations

This study is the first in Ethiopia to compare and report the safety and efficacy of surgical versus medical abortion. The study enrolled adequate numbers of patients in the medical and surgical arms during the study period. Saint Paul’s Hospital Millennium Medical College is the only public institution so far in the country providing D&E for abortion patients, and only recently started this practice, together with the initiation of a family planning fellowship for graduates of an OBGYN residency program. This study demonstrates that D&E can be safely and effectively initiated and practiced in other similar Ethiopian settings.

The study has several limitations. Study subjects were not randomized to abortion methods, which introduces selection bias, as revealed by the proportion of patients from outside Addis Ababa who chose surgical abortion. Our study limited the comparison to cases between the gestational age of 13–20 weeks and cannot be generalized to abortion before 13 or beyond 20 weeks’ gestation. The study is performed in an established academic institution where there is a family planning fellowship program and should not be generalized to other hospitals that lack sufficient training and oversight in surgical abortion. Significantly fewer patients chose surgical abortion during the study period. Further investigation into reasons for this is needed to identify knowledge gaps and make recommendations.

## Conclusion

Our study demonstrates that surgical abortion is a safe and effective alternative to medical abortion in our institution. We hope these results will inform the current national technical guidelines, which currently consider second trimester surgical abortion a backup method only. Instead, surgical abortion should be a first line option for patients, along with medical abortion.

Attempts to expand the family planning fellowship program and train more providers in Ethiopia to perform surgical abortion should be part of any strategy to improve care.
